# Analysis of mental and physical fatigue over the course of a professional English Premier League season in outfield players

**DOI:** 10.5114/biolsport.2024.133001

**Published:** 2025-03-18

**Authors:** Adam Brett, David Rhodes, Sophie Grimson, John Kiely

**Affiliations:** 1 Institiute of Coaching and Performance, Football Performance Hub, School of Sport and Health Sciences, University of Central Lancashire; 2 Brighton and Hove Albion FC, Brighton, United Kingdom; 3 Department of PE and Sports Sciences, University of Limerick, Ireland

**Keywords:** Mental fatigue, Soccer, Monitoring, Cognition, Visual analogue scale

## Abstract

A variety of studies suggest mental fatigue (MF) negatively influences both perceptions of physical fatigue and psychomotor performance. No prior investigation has examined potential changes in player ratings of MF over the course of an English Premier League (EPL) season. This study analysed how MF and PF varied among elite footballers, at both grouped and individual levels, across a 46 match EPL season. Prospective cohort study. Over the course of a 40-week, 151 training sessions and 46 games season, 21 first team male EPL footballers rated both physical fatigue (PF) and MF on a 100-mm visual analogue scale on match day minus-one (M-1). No significant group differences were established for MF (18.9 ± 13.0 vs. 17.8 ± 14.8 vs. 20.8 ± 20.5) and PF (21.4 ± 14.0 vs. 20.9 ± 14.7 vs. 19.9 ± 17.5) (P > 0.05) between early, mid and late season timepoints. However, pervasive individual differences were evident across timescales. Unlike earlier comparable work in netball, this study did not detect group-based changes in MF or PF over a competitive season. However, extensive changes in individual ratings of MF and PF did occur. This study highlights the potential importance of monitoring and managing MF trends within squads of elite footballers. The 100-mm visual analogue scale provides practitioners with a pragmatic tool capable of, in a resource-efficient manner, monitoring MF in EPL squads. Monitoring MF potentially facilitates the customised provision of targeted remediation strategies encompassing (a) training design, (b) individualized recovery and (c) player education relating to MF alleviation and stress management interventions.

## INTRODUCTION

Fatigue is a complex, multidimensional, task-dependent, perceptual phenomenon and definitions of fatigue differ across both academic disciplines and sporting domains [1]. One notable definition, within sports science contexts, describes fatigue as a disabling psychophysiological symptom underpinned by interactions between (i) performance fatigability, the decline in an objective measure of performance over a discrete time period and (ii) perceived fatigability, changes how sensory signals are subjectively experienced [2]. However, the lack of a definitive description of fatigue, its multi-faceted nature and the varied inputs that collectively shape the fatigue experience, conspire to ensure that direct quantification is problematic [2, 3, 4].

Multiple surrogate measures of fatigue are habitually assessed in professional sporting contexts, with practitioners predominantly focusing on surrogates of physical fatigue assessed via various tests such as, for example, metrics derived from countermovement jumps and landings [4]. However, in football contexts, both research and practice largely neglect mental dimensions of fatigue. Consideration of mental fatigue (MF) may, however, be important. Notably, a recent systematic review concluded that MF, induced using various methodologies in various non-elite athlete populations, reduced endurance performance across a range of endurance-related activities [5]. The review concluded by (a) recommending that mentally demanding tasks be avoided before competitions demanding optimized performance and (b) calling for much needed research on MF within elite athlete populations. Similarly, recent findings suggest mounting MF incurs compensatory increases to physical exertion and physical fatigue. Furthermore, the accumulation of MF has previously been associated with declining soccer-specific technical and decision-making performance [6, 7, 8, 9].

Initial, albeit sparse, evidence suggests mental fatigue may be exacerbated following competitive match play. Two recent studies, investigating mental fatigue in professional soccer academy players – acutely, post-match play, and chronically, across a season, found competitive match play increased subjective mental fatigue in the first day post-match (M+1) [10, 11]. In one study mental fatigue was significantly modulated by match result [12]. In the other, MF was modulated by fixture congestion (44 ± 25 AU) [13]. Importantly, however, it remains unclear how MF modulates in tandem with PF and whether MF persists for multiple days, post-match, and/or whether residual MF accumulates in elite footballing populations over the course of specific competitive periods.

Additionally, whether mental fatigue drives declining physical performance remains controversial. Recent contradictory findings failed to replicate Marcora’s landmark demonstration of the negative influences of MF on cycling performance [2, 3], and on physical performance during small-sided games [13]. Furthermore, a recent publication concluded almost half of the studies within this literature did not exhibit performance declines associated with mounting MF [14].

The issue of whether MF varies in elite athletes over the course of a competitive season remains unresolved. Only two studies have been conducted using athletes performing at elite or sub-elite levels. Interestingly, these studies arrived at contrasting conclusions. In elite netball mental fatigue significantly increased, whereas in elite academy footballers mental fatigue decreased, in late-, compared to early- or mid-, season [15]. Notably, whether MF is likely to be more or less susceptible to change in older, more seasoned and experienced senior English premier league (EPL) players, than in younger and less experienced Academy players, remains unclear. Moreover, prior investigations were conducted in contexts where the number of competitive matches played was substantially less than that habitually experienced by EPL players. Abbott et al., [10] studied MF over 37 matches, whereas Russell et al [15] studied MF over 17 matches. In contrast, EPL footballers involved in European competitions may play up to 60 matches per season. Furthermore, EPL squads involved in European competitions may participate in two games per week during peak competitive season; thereby heightening the potential for accumulating MF and PF during these periods. These context-specific demands underline the importance of exploring fatigue characteristics within this highly specialised cohort.

Despite the potential relevance of MF to competitive performance, prior research remains inconclusive. Driven by this lack of experimental insight, previous authors have suggested that to enhance our understanding of how mental fatigue develops in athletes, future research should focus on tasks and demands specific to the athlete [16, 17]. In addressing this deficit, the objectives of this investigation were to examine how mental fatigue varies in elite EPL footballers, at both grouped and individual levels, over the course of the English Premier League season, using previously validated subjective tools [10].

## MATERIALS AND METHODS

Ethics was approved by the BAHSS2 ethics panel at the University of Central Lancashire (UK). All participants provided informed consent.

Data was collected from 21 first team professional male English Premier League footballers (height: 180.9 ± 9.3 cm; Weight: 77.8 ± 8.8 kg; age: 25.3 ± 3.8 yr) over a 40-week season (2020–2021). Table 1 identifies the 3 periods of data collection, number of timepoints within these phases, average number of training sessions and games completed over the EPL season. The season was broken down in to 3 phases due to MF previously being found to be sensitive to phases of season [14]. Furthermore, a phased approach was chosen as the research was conducted in an applied environment, where fixture congestion is highest in the middle of the season over the Christmas period – this allowed for similar comparatives within timepoints for matches played (15 v 15 v 16).

**TABLE 1:** The breakdown of training sessions and matches across the 2020–2021 season

	Early (21.08.20–12.12.20): 17 weeks	Middle (19.12.20–09.02.20): 10 weeks	Late (12.02.20–22.05.20): 15 weeks
Number of Timepoints (Matches)	15	15	16
Average number of training sessions	3.89	3.38	3.86
Average number of games per week	1.11	1.63	1.0

This investigation was designed as a prospective cohort study. Players took part in 151 training sessions and 46 matches across the season. The breakdown of the subsequent training and matches is displayed in Table 1. All players were familiarised with the subjective measure to assess mental fatigue (MF) and physical fatigue (PF) prior to the season commencement. On the day of administering the questionnaire, the questionnaire was completed prior to training commencement during morning monitoring (between 9:00 and 9:30 AM). Player data was excluded from analysis if players were injured or ill.

**FIG. 1:**
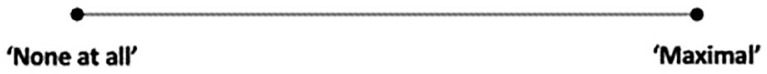
Example of 100 mm VAS scale.

Subjective ratings of mental fatigue (MF) and physical fatigue (PF) were collected on the morning of match day minus 1 (MD-1) at 46 timepoints across the season. MD-1 allowed a consistent window where the medical department assessed player status to plan and propose recommendations to the coaching staff and was part of the normal weekly routine for the players. Subjective ratings of MF and PF were assessed using a visual analogue scale (VAS), previously demonstrated as sensitive to changes in MF in athletic populations [10, 16, 17]. MF was defined as ‘reduced ability to perform cognitively demanding activity and characterised by tiredness and lack of energy’ [3]. PF was defined as a ‘reduced capacity to perform maximally, solely due to physical tiredness (e.g. muscle soreness)’. The VAS is displayed below in Figure 1. It was represented by a 100 mm line, and completed by pen and paper, and measured. To reduce the potential for a social influence response bias, VAS scores were completed in complete isolation. MF and PF monitoring is completed as part of the clubs normal daily monitoring. All players were familiarised and educated with the scales, definitions and protocols associated with this monitoring during the pre-season period before data collection began, to ensure clarity and understanding.

Data analysis was completed using SPSS (SPSS Version 26.0). Normal distribution was considered if the Shapiro-Wilk test was p > 0.05. Data for MF and PF were analysed across three phases of the season (Table 1). A one-way repeated measures analysis of variance (ANOVA) assessed differences in pre-match MF and PF across the early (15 time points), mid (15 time points) and late (16 time points) season phases. Data was assessed in alignment with previously published investigations within mental fatigue in elite soccer [14].

For both MF and PF, group-level changes and individual within-player variability was analysed. The predicted linear trend was calculated for both MF and PF separately utilizing both the early and then mid-season phase values. Subsequent weeks scores, falling outside the typical range could then be identified. Mid-season values were compared to the early season trend, and late-season values were compared to the mid-season trend. Findings were interpreted using magnitude-based inferences [19]. Uncertainty in the effect was expressed as 90% confidence limits, with likelihoods that the true value of the effect represented substantial or trivial changes expressed as possible (25–75%), likely (75–95%), very likely (95–99%) and most likely (> 99.5%) [24]. The smallest worthwhile change was determined at 0.2 within standard deviation. A paired-samples t-test was used to determine differences between PF and MF across questionnaires. Cohens d was utilised to determine strength of differences obtained within the t-test (0.1 = small, 0.3 = medium, and 0.5 = large) [20]. Statistical significance was determined at p < 0.05.

## RESULTS

**FIG. 2:**
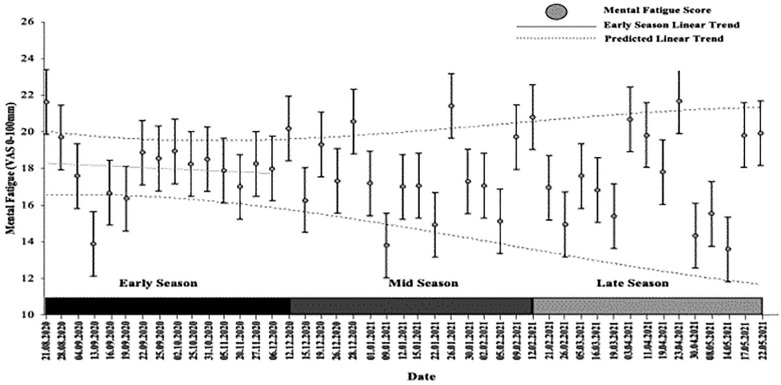
Example analysis of the average group modelled early season trend vs. mid and late season for MF. Values and error bars beyond the dashed lines represent a true change that is above 90% likelihood.

The group analysis of MF and PF data for the first team squad at an EPL club across the 2020/21 season is presented in Figure 2.

One-way repeated measures ANOVA revealed no main effect for time (*f*_(2,34)_ = 0.545, p = 0.585, *h*^2^_partial_ = 0.031), and no changes in MF between early, mid, and late season (18.9 ± 13.0 vs. 17.8 ± 14.8 vs. 20.8 ± 20.5). The modelled early-season and midseason phase trend analysis (Figure 2) revealed no 90% likely true changes in MF when compared to remaining time points. The modelled mid-season trend analysis revealed no 90% likely true changes in MF, when compared to remaining time points.

One-way repeated measures ANOVA revealed no main effect for time (*f*_(2,34)_ = 0.569, p = 0.572, *h*^2^_partial_ = 0 .032), and no changes in PF between the early, mid, and late season (21.4 ± 14.0 vs. 20.9 ± 14.7 vs. 19.9 ± 17.5). Modelled early-season phase trend analysis, when compared to remaining time points across the season, revealed 3 90% likely true changes in PF, including a significant increase on dates 02.11.2021 (round 10) and 21.05.2021 (round 38), and a significant decrease on the 5.11.21 (round 11). Modelled mid-season trend analysis when compared to remaining time points revealed 1 90% likely true change in PF, including a significant increase on the 21.05.21.

Modelled early season trend analysis, on an individual level, when compared to mid-season and late season, were 90% likely true for MF and PF. Modelled mid-season trend analysis on an individual level, when compared to the late season, were 90% likely true. Individual responses to the modelled early season trend vs. mid and late season phases are illustrated in Table 2.

**TABLE 2:** The individual responses to the modelled early season trend vs. mid and late Season phases.

		Early-Mid Phase		Mid-Late Phase	
Dimension		MF	PF	MF	PF
**Player Identifier**	1	-	-	↑*	↑*
	2	↑*	-	↑*	-
	3	-	-	↑*	-
	4	↑*	↑*	↑*	↑*
	5	↓*	-	-	-
	6	↑*	↑*	↑*	↑*
	7	↑*	↑*	↓*	↓*
	8	↑*	↑*	↑*	↑*
	9	↑*	↑*	↓*	-
	10	-	↑*	↓*	↓*
	11	↓*	↓*	-	-
	12	/	/	-	-
	13	-	↑*	↑*	-
	14	/	/	↑*	↑*
	15	↓*	↑*	↑*	↑*
	16	↑*	↑*	↓*	↓*
	17	-	↑*	/	/
	18	/	/	↓*	↓*
	19	↓*	↓*	↑*	-
	20	↑*	↑*	↑*	↓*
	21	-	-	↓*	↓*

* MF = Mental Fatigue; PF = Physical Fatigue. A change with the chances > 90% is very likely or decisive and is indicated with a ↑, ↓ and <–>. An increase in MF during the retrospective phases in comparison to the modelled early season trend are demonstrated with an ↑ symbol. A decrease in MF during the retrospective phases in comparison to the modelled early season trend are demonstrated with a ↓ symbol. A maintenance in MF during the retrospective phases in comparison to the modelled early season trend with a <–> symbol. A – symbol represents any changes with a chance of < 90% likelihood.

As illustrated in Table 3, in comparison to modelled early in-season trend, during the mid-season phase, MF increased in 8, decreased in 4, and remained the same in 6, of 18 participants. During the late season phase, MF increased in 11, decreased in 5, and remained the same in 2 of 18 participants. In comparison to the mid-season trend, MF increased in 11, decreased in 6 remained the same in 3 of 20 participants.

In comparison to the modelled early in-season trend, during the mid-season phase, PF increased in 11, decreased in 2 and remained the same in 5 of 18 participants. During the late season phase, PF increased in 8, decreased in 2 and remained the same in 8 of 18 participants. In comparison to the modelled mid-season trend analysis, PF increased in 6, decreased in 6 and stayed in the same in 7 of 20 participants. Interestingly, across all questionnaire’s administered, average PF scores were higher (21.85 ± 19.31) than MF score (18.76 ± 17.23) (T_(1212)_ = -7.095, *p* = 0.000, *d* = -0.20).

The individual analysis for MF and PF across the 20–21 season in an elite EPL team are presented in Table 3.

**TABLE 3:** The overall 90% likely true changes in MF and PF across the season in comparison to the early-season trend and mid-season trend.

	Early Mid			Mid-Late		

	Increase	Decrease	Stayed the same	Increase	Decrease	Stayed the same
MF	8	4	6	11	6	3
PF	11	2	5	6	6	8

## DISCUSSION

This paper examined how mental fatigue varied over the course of the EPL season at both the level of the squad and the individual player. Findings revealed mental fatigue did not change, at the squad level across the season. Interestingly, this contradicts the findings by Russell et al. [22] who reported both mental and physical fatigue increasing across a netball season. The Russell paper however investigated development-level netball players whereas this investigation examined responses in seasoned professionals; thereby suggesting the potential for some accumulative habitual mental resilience from years of exposure. In addition, there is potential for greater support and remedial psychological provisions within elite professional soccer contexts, when compared to development-level squads. Interestingly, grouped physical fatigue (PF), did increase in the last phase of the season. Potentially illustrating the heterogeneity between sample groups, with professional soccer players demonstrating greater individual variability. Notably, these results highlight that different dimensions of the fatigue phenomenon may change independently. An observation that should be explored in greater detail.

In the only prior comparable soccer-specific investigation Thompson et al. [11] demonstrated that mental fatigue increased in a group of elite academy players across a season, and suggested MF was likely to be greatest in those experiencing higher seasonal training and playing demands. In contrast, Russell et al. [15] monitoring mental fatigue in elite netballers across two seasons, observed lowest levels of MF in the last week of competition. This observation was interpreted as indicating that athlete’s anticipation of an upcoming training and competitive break alleviates MF [17, 22]. Our results, however, illustrated that MF varies on an inter-individual basis, across seasonal timeframes. Both studies do, however, suggest that MF is an independent measure of player well-being and, as such, should be regularly monitored. This research builds on previous work demonstrating that mental fatigue negatively impacts diverse facets of sports performance [2], soccer-specific skills [8] and decision making [9]. Accordingly, the findings of this paper provide further support for monitoring of both biological and psychological aspects of fatigue within elite soccer squads.

The Russell group established that MF was higher during Netball training and pre-competition camps than during competition periods [23] and that, across the course of a 16-week pre-season phase, higher MF ratings were observed later in the season [15]. The shared variance between physical fatigue and mental fatigue during elite netball matches was 14.3% [17], subsequently illustrating that mental and physical fatigue are largely different constructs which although interactive, should be considered as separate. The findings of our study similarly revealed significantly different physical and mental fatigue across all phases of the season, suggesting athlete differentiation of the two parameters of fatigue. Interestingly, physical fatigue was always reported as higher than mental fatigue. Subsequently, we suggest these results emphasize the importance of athlete wellbeing, thereby highlighting the need for more effective means of educating players and staff, and the need to develop more evidence-led interventional strategies.

Analysis of MF at the level of the individual player revealed extensive inter-individual variability, in the mid phase versus the early season trend as both players and performance staff perceived MF as negatively impacting sporting performance [16] The results of this paper reinforce the importance of further exploring the impact of MF at the level of the individual player.

The MF experienced, at both an inter- and intra-individual level, may be modulated by multiple shaping influences, including:

i. Innate predispositions and distinct histories of prior MF exposures among players [24, 25, 26]
ii. Individual differences in players cognitive and psycho-emotional stress coping capacities and recovery strategies [14]
iii. Individual differences in predispositions and perceptions of soccer-specific (results, performance, selection security, injury anxiety) and non-specific (media, financial and general life) stressors [10, 27]
iv. Distinct personal histories, including sporting and life experiences [27]
v. Access to family, friends and/or organisational support networks [28]

The multi-faceted nature of this inter-individuality suggests that soccer players may develop mental fatigue, both acutely and chronically, due to diverse innate and environmental influences. The extent of this heterogeneity supports the utility of monitoring mental fatigue to enable individual personalization of recovery interventions in elite soccer environments.

### Practical implications

Conventionally, within professional soccer contexts, physical fatigue is routinely assessed and tracked. Mental fatigue, in contrast, is typically neglected. Nevertheless, the evidence presented here illustrates that MF, at both squad and player levels, changes across the span of an EPL season. This highlights the importance of monitoring MF at an individual level.

Monitoring MF over the course of the season potentially facilitates the customised provision of targeted enhancement strategies encompassing (a) training management, (b) individualized recovery interventions and (c) player education specifically relating to MF and stress management strategies.

Additionally, this study supports the use of a 100-mm VAS tool as a sensitive mental fatigue monitoring tool that practitioners can use to evaluate mental fatigue. Notably this tool was easily applied in EPL settings and, due to its ease of application, did not disrupt existing game or training day practices and thereby facilitated player buy-in to the monitoring process.

In the future subjective VAS ratings may prove most informative when combined with other subjective and/or objective metrics, such as those already deployed professional football contexts. For example: measures of external load, such as in-game GPS-derived metrics and jump kinetics and/or surrogate measures of internal load, such as RPEs, heart rate variability and hormonal biomarkers.

In short: the utility of isolated metrics may be fundamentally limited. The consideration of multiple relevant metrics may better inform decision-making processes. VAS ratings, especially given their practical utility, may be worthwhile inclusions in such data gathering processes. Clearly, however, more research is needed.

### Limitations

The visual analogue scale utilised in this investigation has some limitations, as previously noted by others [10]. Key among these limitations is the inescapable fact that the accuracy and relevance of VAS is dependent on players honestly rating their subjective MF scores. Furthermore, the physical, cognitive and psycho-emotional demands of association football may vary extensively, under the influence of multiple personal and situational modulating factors. Player roles and positions within a team, for example [30]. Such influences were not accounted for in this study. Similarly, senior professional players with extensive experience may have developed coping strategies and some “mental resilience” to the external stressors associated with matchplay. Again, this potential modulating factor was not considered within this investigation. Similarly, whether MF accumulation differs between male and female footballers remains unexamined.

### Practical Implications

– A variety of studies suggest mental fatigue influences diverse dimensions of physical performance; yet mental fatigue is not routinely assessed or tracked in elite soccer settings
– Group-based analysis of MF may mask extensive inter-individual, player-specific responses to the challenges imposed during an EPL season
– MF can be routinely assessed using time- and resource-efficient assessment tools
– Information on individual player-specific modulations in MF may be deployed to inform customized remedial and/or recovery strategies

### Future direction

The phenomenon of fatigue can be sub-divided into a number of distinct sub-capacities.

Currently, it remains unclear how these capacities mutually comodulate over the course of a competitive sports season. Furthermore, it remains unclear whether and how distinct sub-capacities (such as MF) can be specifically monitored and beneficially targeted by focused training interventions to foster enhanced fatigue-resilience.

Subsequently, future research should focus on practically relevant issues, such as:

i. The evolution of monitoring tools to assess MF, training protocols to enhance MF resilience, and remedial strategies to alleviate the negative consequences of MF
ii. The influence of contextual factors (such as: coach behaviours, travel demands, results, media pressures, management change, fixture congestion and match outcomes) on mental fatigue
iii. The determination of whether MF is primarily amplified by the pressures and stressors surrounding life as a professional footballer and/or the physical, cognitive and emotional rigors of match play

## CONCLUSIONS

A variety of studies suggest mental fatigue influences diverse dimensions of physical performance (attention, physical endurance) [7] and sport-specific psychomotor performance [29]. Nevertheless, mental fatigue is not routinely assessed in elite soccer contexts [16]. The findings presented here suggest players perceive mental and physical fatigue as distinct phenomena.

At a group level mental and physical fatigue did not change significantly across the three (early, mid and late) phases of the season. Notably, individual differences were evident, and pervasive, across the season in elite senior soccer players. Suggesting, subsequently, that an in-depth knowledge, facilitated by ongoing monitoring, will enhance coaching and technical staff’s ability to understand, track and design interventions to enhance players resilience to mental fatigue. Further research, to confirm the extent of the physical demand of match-play and the individual variability within player perception, could be incorporated to investigate the correlation between mental fatigue with differing levels of physical exertion and different competitive demands.

Ultimately a clearer, more detailed, understanding of how individual players respond to the demands and stresses of a full EPL season will enable the development of bespoke, personalised strategies to enhance the monitoring and remediation of MF imposed across the competitive season.
